# Antimicrobial Testing: Advancing Methodologies and Addressing Global Challenges

**DOI:** 10.3390/microorganisms13102370

**Published:** 2025-10-15

**Authors:** Suresh G. Joshi

**Affiliations:** 1 Department of Surgery, College of Medicine, Drexel University, Philadelphia, PA 19102, USA; sgj24@drexel.edu; 2 School of Biomedical Engineering, Science, & Health Systems, Drexel University, Philadelphia, PA 19104, USA

An increasing prevalence of antimicrobial resistance (AMR) represents a formidable threat to global health, demanding the urgent evolution of diagnostic methods and therapeutic strategies [1,2,3,4]. Central to this endeavor is “antimicrobial testing, a foundational discipline that informs treatment guidelines, monitors resistance patterns, and evaluates the performance of emerging antimicrobial agents [5,6].

Antimicrobial Testing, the Special Issue of *Microorganisms*, now compiled as a standalone volume, presents a selection of high-quality articles that reflect the current state of antimicrobial testing—from methodological refinements in susceptibility essays to evaluations of alternative therapies. These contributions explore not only classical antibiotic testing approaches but also innovations such as high-throughput screening systems, biofilm-resistance profiling, phage therapy validation, and testing against multidrug-resistant clinical isolates [7,8].

Many of the articles underscore a critical challenge in the field: bridging in vitro results with in vivo efficacy. For instance, testing within biofilm models or in polymicrobial environments more accurately represents the clinical setting, where pathogens behave in complex, often unpredictable ways. The need for harmonized protocols that go beyond MIC and disk diffusion toward dynamic, system-level analyses is a recurring theme in this volume [8].

The aim of this book is to provide a valuable reference for researchers, clinicians, pharmaceutical developers, and policymakers involved in infectious diseases, microbiology, and drug development. It emphasizes the urgency of innovation in antimicrobial testing as a core component of global strategies to mitigate AMR and improve patient care outcomes.

I extend my sincere thanks to the contributing authors, the peer reviewers for their rigorous assessments, and the editorial team at MDPI *Microorganisms* for their unwavering support. This volume reflects a collaborative scientific spirit that is essential to addressing one of the defining biomedical challenges of our time.

## 1. Foreword

Antimicrobial resistance (AMR) has emerged as a silent pandemic, threatening to erode decades of progress in modern medicine [9]. The effectiveness of antimicrobial therapy depends fundamentally on accurate and clinically relevant antimicrobial testing, which serves as the bridge between bench science and bedside care [10].

This book—assembled from a Special Issue of *Microorganisms*—provides readers with a timely, rigorous, and diverse selection of research on antimicrobial testing. From refined susceptibility assays and biofilm models to evaluations of phage therapy and novel drug compounds, the chapters herein reflect the cutting edge of a field tasked with nothing less than safeguarding the future of infection management.

As editor, it is my privilege to introduce this collection, and I hope it will serve as a valuable resource for the broader scientific community committed to curbing AMR through innovation, standardization, and collaboration.

Dr. Suresh G. Joshi

Drexel University College of Medicine, Philadelphia, PA, USA

## 2. Acknowledgments

I would like to express my heartfelt appreciation to all contributing authors for their high-quality research and thoughtful contributions to this Special Issue. I also acknowledge the critical role played by the editorial and production teams at *MDPI Microorganisms*, who ensured the seamless coordination and publication of this collection. Ms. Jennifer Shi, journal development editor, provided valuable editorial help in inviting articles and following up the authors’ commitments in this direction. Special thanks are due to the peer reviewers whose feedback upheld the scientific rigor of each article.

This book is dedicated to the global community of microbiologists, clinicians, and researchers working tirelessly to improve antimicrobial testing and combat antimicrobial resistance for the benefit of current and future generations.

## 3. Back Cover Description

### About This Book

This book is a collection of peer-reviewed articles originally published in the Special Issue *Antimicrobial Testing* of journal *Microorganisms* (MDPI). It brings together cutting-edge research on antimicrobial susceptibility assays, resistance mechanisms, alternative therapeutic testing, and methodological innovations critical to the understanding of diagnosis and treatment of infectious diseases. The volume serves as a reference for microbiologists, infectious disease specialists, pharmaceutical researchers, and global health professionals engaged in the fight against antimicrobial resistance.
